# Extent of Fentanyl Accumulation Following Multiple Doses of Fentanyl Buccal Tablet 400 µg in Healthy Japanese Volunteers

**DOI:** 10.1111/j.1753-5174.2008.00008.x

**Published:** 2008-09

**Authors:** Mona Darwish, Kenneth Tempero, John G Jiang, Philip G Simonson

**Affiliations:** * Cephalon, Inc.Frazer, PA, USA; † 1901 Lake Road, Wayzata, MN, USA

**Keywords:** Fentanyl Buccal Tablet (FBT), Pharmacokinetics, Japanese

## Abstract

**Objective:**

This study was conducted to characterize the pharmacokinetics, including extent of accumulation, and safety and tolerability of fentanyl following multiple doses of fentanyl buccal tablet (FBT) in healthy Japanese volunteers.

**Methods:**

Healthy Japanese adults received 10 successive doses of open-label FBT 400 µg at 6-hour intervals. Naltrexone was given to minimize the opioid effects of fentanyl. FBT was placed above a molar tooth between the gum and cheek. Peak serum fentanyl concentration (C_max_), time to C_max_ (t_max_), and area under the serum fentanyl concentration-time curve from 0 to 6 hours (AUC_0–6_) were summarized using descriptive statistics. Accumulation ratio was calculated as C_max_ for dose 10/C_max_ for dose 1, and was calculated similarly for AUC_0–6_.

**Results:**

Fourteen volunteers (mean age 33 years) were enrolled, and 13 completed the study. After doses 1 and 10, respectively, mean (SD) C_max_ was 1.70 (0.49) ng/mL and 1.97 (0.42) ng/mL, AUC_0–6_ was 4.46 (1.14) ng·h/mL and 6.81 (0.90) ng·h/mL, and median (range) t_max_ was 50 (30–110) minutes and 30 (15–120) minutes. Following 10 successive doses, systemic exposure (AUC_0–6_) was 55% higher than after dose 1, and C_max_ was 23% higher. Steady state was achieved within 3 days of dosing at 6-hour intervals, i.e., prior to dose 10. The most frequent adverse events (AEs) were somnolence (N = 9), decreased oxygen saturation (N = 4), headache (N = 3), application-site pain (N = 8), application-site erythema (N = 6), and application-site reaction (N = 5). All AEs were mild or moderate.

**Conclusions:**

Following administration of FBT at 6-hour intervals to healthy Japanese volunteers, at steady state, fentanyl exposure was higher by 55% (AUC_0–6_) and 23% (C_max_) than after a single dose of FBT. Adverse events were mild or moderate.

## Introduction

Fentanyl buccal tablet (FBT; *FENTORA*®, Cephalon, Inc., Frazer, PA, USA) is approved for marketing by the US Food and Drug Administration for the management of breakthrough pain in patients with cancer who are already receiving and who are tolerant to around-the-clock opioid therapy for their underlying persistent cancer pain [1–3]. FBT employs OraVescent® technology to enhance the rate and extent of absorption of fentanyl across the buccal mucosa [4]. The pharmacokinetic profile of FBT has been examined in several studies, primarily involving healthy white volunteers [5–9]. The current study was conducted to support the submission of a new drug application for FBT in Japan. The objectives of this study were to characterize the pharmacokinetics of fentanyl, including the extent of accumulation, and to assess the safety and tolerability of fentanyl following multiple doses of FBT 400 µg in healthy Japanese volunteers.

## Methods

### Study Population

Healthy, adult Japanese volunteers residing in the United States who were aged 20 to 55 years, had lived fewer than 10 years outside of Japan, and had a body mass index of 17.6 to 29 kg/m^2^were eligible for this study. Volunteers agreed to refrain from using tobacco products throughout the study. Women of childbearing potential were required to practice abstinence or use a reliable form of contraception for 14 days prior to screening and throughout the study.

Exclusion criteria included a history or presence of clinically significant concomitant disease, including clinically significant laboratory, electrocardiogram (ECG), or physical examination results at screening; history or presence of alcoholism or drug abuse; hypersensitivity or idiosyncratic reaction to fentanyl or related compounds; history of frequent or widespread bilateral canker sores or current oral pathology; or participation in another clinical study within the previous 30 days. Volunteers were also excluded if they had smoked >10 cigarettes per day within 3 months, taken prescription medications within 14 days or over-the-counter medications within 7 days, or used drugs known to be strong inhibitors or strong inducers of cytochrome P450 (CYP) enzymes within 10 and 30 days, respectively, prior to administration of the first dose of study drug. In addition, women were excluded if they were pregnant or lactating.

### Study Design

This study was conducted at a single center (Radiant Research, Honolulu, HI). The protocol was approved by the Quorum Review, Inc., Institutional Review Board. All volunteers provided written informed consent.

Volunteers received 10 successive single doses of open-label FBT 400 µg, administered at 6-hour intervals. Because healthy volunteers are not opioid tolerant, the volunteers in this study also received oral naltrexone 50 mg (Barr Pharmaceuticals, Inc., Pomona, NY) 15 hours and 3 hours before the first FBT dose, and every 12 hours after the first dose (for a total of 7 doses) to minimize the opioid receptor–mediated effects of fentanyl. Coadministration of naltrexone with fentanyl would not be expected to affect the pharmacokinetics of fentanyl because fentanyl is a substrate of CYP3A4 [10], and naltrexone is not an inhibitor or inducer of CYP3A4 [11].

Volunteers self-administered FBT by placing a single tablet buccally above a molar tooth and allowing it to disintegrate for 10 minutes. If any portion of the tablet remained after 10 minutes, the volunteer gently massaged the adjacent cheek area for 5 minutes, at which time study center personnel visually inspected the volunteer's mouth to note whether tablet particles were present. (It should be noted that massage is no longer recommended in the prescribing information [2].) Any remaining material was allowed to disintegrate. Volunteers refrained from drinking any liquids until complete disappearance of the tablet residue, and remained seated for 2 hours following administration of each of the first 3 FBT doses.

### Dwell Time Assessment

Buccal dwell time, the time elapsed between placement of FBT and complete disappearance of tablet residue, was verified visually and recorded by study personnel.

### Sample Collection

Venous blood samples (5 mL each) were collected for determination of fentanyl serum concentrations. Collection times were just before placement of each dose and 3, 6, 9, 12, 15, 20, 25, 30, 40, 50, 60, 70, 80, 90, 100, and 110 minutes, and 2, 4, and 6 hours after placement of dose 1. Samples were also collected 15, 30, 40, 50, 60, 70, 80, 90, 100, and 110 minutes, and 2, 4, 6, 10, 12, 18, 24, and 32 hours after placement of dose 10.

Blood samples were kept at room temperature until they fully clotted. They were then centrifuged at 2,500 revolutions per minute at 4°C for 15 minutes to separate the serum, which was stored in polypropylene containers at or below −20°C until assayed.

### Analytical Methods

Fentanyl concentrations were quantitated in serum samples using a validated assay for high performance liquid chromatography with tandem mass spectrometry (HPLC-MS/MS; PE Sciex API 3,000, API 365, and API III Plus with an ESI interface). The analyte and internal standard (d_5_-fentanyl) were extracted from serum under basic conditions. Serum extracts were evaporated to dryness and reconstituted before injection onto the HPLC-MS/MS. Detection was accomplished using multiple-reaction monitoring in positive ion mode. The linear range for the assay was 10 to 5,000 pg/mL (0.01–5.0 ng/mL). The lower limit of quantitation was set at 50 pg/mL (0.05 ng/mL), and fentanyl concentrations below this level were given a value of 0.0 in the computation of mean concentration values. For quality control samples, interbatch precision (% coefficient of variation) was ≤4.6% and accuracy ranged from 95.5% to 102.0%.

### Pharmacokinetic Analysis

For doses 1 and 10, maximum serum fentanyl concentration (C_max_), time to C_max_ (t_max_), and area under the serum fentanyl concentration-time curve (AUC) from time 0 to 6 hours (AUC_0–6_) were determined. Additional pharmacokinetic parameters determined for dose 10 included minimum serum fentanyl concentration (C_min_), AUC from time 0 to last quantifiable serum concentration (AUC_0–last_), and AUC from time 0 extrapolated to infinity (AUC_0–∞_). Trough concentrations (C_trough_) were analyzed in blood samples obtained immediately preceding each dose. C_max_, C_min_, and t_max_ were obtained by inspection. AUC_0–6_was obtained by linear trapezoidal summation from time 0 to 6 hours post dose, and AUC_0–last_ was obtained by linear trapezoidal summation from time 0 to the last quantifiable concentration after the last dose. AUC_0–∞_ was obtained as AUC_0–last_ + C_last_/λ_z_, where C_last_ is the last quantifiable serum fentanyl concentration and λ_z_ is the terminal elimination rate constant. The half-life (t_½_) could not be calculated after dose 1 because there were insufficient analysis time points, i.e., only up to 6 hours. The t_½_ and λ_z_ were determined from the terminal phase of the last dose administered. In addition, the accumulation ratio was calculated as the AUC_0–6_ ratio for dose 10 to dose 1 and as the C_max_ ratio for dose 10 to dose 1. Pharmacokinetic parameters were determined from concentration-time data using noncompartmental methods with WinNonlin® Professional software Version 4.1 (Pharsight Corporation, Mountain View, CA).

### Safety and Tolerability Assessments

Clinical laboratory tests, 12-lead ECG, and physical examinations including vital signs were performed at screening and at study completion or early termination. Serial vital signs were measured before and through 6 hours after the first tablet placement, and prior to all blood sampling times. Continuous pulse oximetry was conducted, though not recorded, for the first 4 hours post dose and whenever the volunteers attempted to sleep during the study in the clinic. In addition, the oral mucosa was examined by the investigator 1 hour after tablet placement; any abnormality or untoward or uncomfortable sensations in the mouth were recorded as adverse events (AEs). Other AEs observed by study personnel or reported by volunteers also were recorded.

### Statistical Analyses

Descriptive statistics were used to summarize the calculated pharmacokinetic parameters. Unless otherwise stated, statistical tests were 2-sided with an α level of 0.05. The accumulation ratio was calculated as AUC_0–6_ for dose 10/AUC_0–6_ for dose 1, and as C_max_ for dose 10/C_max_ for dose 1. To assess the attainment of steady state, analysis of variance (anova) was performed on the natural log of pre-dose concentrations for dose 8, 9, and 10, as well as the 6-hour post-dose concentration after the last dose. Both the pharmacokinetic population and safety population were defined as all volunteers who received FBT. It was planned that 14 subjects would be enrolled such that, after dropouts, 10 subjects would complete the study. The planned final sample size of 10 subjects was not based on a statistical power calculation.

## Results

### Study Population

Fourteen healthy Japanese adults (7 men, 7 women) aged 20 to 50 years were enrolled in the study (Table 1) and were included in both the pharmacokinetic and safety populations. One volunteer was discontinued after the eighth FBT dose because of AEs; thus, 13 volunteers completed the study.

**Table 1:** Demographic variables

	N = 14
Age, year (mean [SD])	33 (8)
Sex, female (N [%])	7 (50)
Weight, kg (mean [SD])	57.0 (9.1)
Height, cm (mean [SD])	163.9 (9.2)
Body mass index, kg/m^2^ (mean [SD])	21.2 (2.1)

SD = standard deviation.

### Pharmacokinetic Results

**Figure 1:**
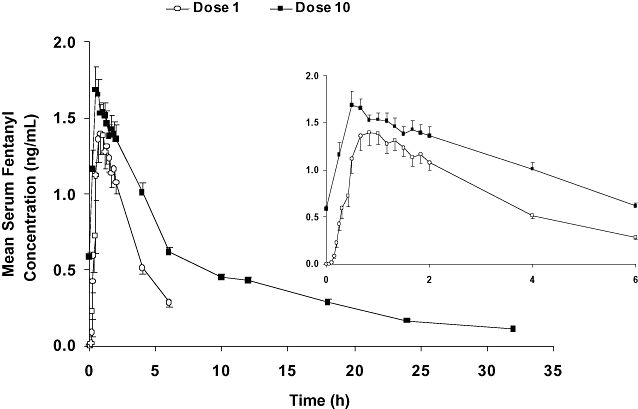
Mean (standard error of the mean) serum fentanyl concentrations after administration of 1 dose and 10 doses of fentanyl buccal tablet 400 µg at 6-hour intervals (inset shows the first 6 hours).

The serum fentanyl distribution/elimination pattern was triphasic after dose 10 (Figure 1). Pharmacokinetic parameters after dose 1 and dose 10 are presented in Table 2. Median t_max_ occurred 50 minutes after dose 1 and 30 minutes after dose 10. Mean C_max_ and systemic exposure (AUC_0–6_) were higher after dose 10 than after dose 1. After dose 10, the AUC_0–last_ and AUC_0–∞_ values were similar (Table 2). Accumulation of serum fentanyl from dose 1 to dose 10 was shown by the calculated accumulation ratios for AUC_0–6_ (1.55) and C_max_ (1.23). The mean t_½_ after dose 10 was 10.29 hours (Table 2). Mean (SD) trough values after dose 7, 8, and 9 were 0.71 (0.17), 0.68 (0.15), and 0.58 (0.12) ng/mL, respectively, and the mean (SD) value at 6 hours after dose 10 was 0.62 (0.10) ng/mL. Steady state was achieved within 3 days of dosing at 6-hour intervals, i.e., prior to dose 10 (Table 3).

**Table 3:** Statistical evaluation of steady state after buccal administration of fentanyl buccal tablet 400 µg every 6 hours for 10 doses

		Steady state *P*value*				
Sample time	N	Least squares mean (ng/mL)	Sample time effect	vs. predose 9	vs. predose 10	vs. postdose† 10
Predose 8	14	0.6877	0.0007	0.3293	0.0002	0.0057
Predose 9	13	0.6549	—	—	0.0029	0.0604
Predose 10	13	0.5586	—	—	—	0.2162
Postdose 10†	13	0.5947	—	—	—	—

* Equivalence across listed samples was assumed if *P* ≥ 0.05.

† Sample taken 6 hours following dose 10.

Note:Pre-dose concentration analysis was performed with an anova model, ln(concentration) = subject + sample time, where subject was a random effect and sample time was a fixed effect.

**Table 2:** Pharmacokinetic parameters following administration of 1 dose of fentanyl buccal tablet 400 µg and 10 doses at 6-hour intervals

	Dose 1	Dose 10
	(N = 14)	(N = 13)
Parameter	Mean (SD)	Mean (SD)
C_max_ (ng/mL)	1.70 (0.49)	1.97 (0.42)
t_max_ (min)*	50 (30, 110)	30 (15, 120)
AUC_0–6_ (ng·h/mL)	4.46 (1.14)	6.81 (0.90)
Accumulation ratio,† C_max_	NA	1.23 (0.53)
Accumulation ratio,† AUC_0–6_	NA	1.55 (0.35)
AUC_0–∞_(ng·h/mL)	NA	16.09 (2.26)
AUC_0–last_ (ng·h/mL)	NA	14.41 (1.83)
C_min_ (ng/mL)	NA	0.58 (0.11)
λ_z_ (1/h)	NA	0.07 (0.01)
t_½_ (h)	NA	10.29 (1.53)

* t_max_ is presented as the median (range).

† For multiple versus single dosing, calculated as C_max_ dose 10/C_max_ dose 1 and as AUC_0–6_ dose 10/AUC_0–6_ dose 1.

C_max_ = maximum serum fentanyl concentration; t_max_ = time to C_max_; AUC_0–6_ = area under the serum fentanyl concentration-time curve (AUC) from time 0 to 6 hours; AUC_0–∞_ = AUC from time 0 to infinity; AUC_0–last_ = AUC from time 0 to the time of the last quantifiable serum fentanyl concentration; C_min_ = minimum serum fentanyl concentration; NA = not applicable; λ_z_ = terminal elimination rate constant; t_½_ = elimination half-life.

### Buccal Dwell Time

The recorded dwell time of FBT in the buccal cavity ranged from 9 to 60 minutes.

### Safety and Tolerability

As stated previously, naltrexone was administered to these healthy volunteers to minimize the opioid effects of fentanyl. Fourteen volunteers had at least 1 AE, all of which were mild or moderate. No serious AEs occurred during the study. One volunteer was discontinued from the study after administration of FBT dose 8 and naltrexone dose 6 because of nausea and vomiting considered by the investigator to have been related to study drug.

The most frequently reported AEs (incidence ≥10% of volunteers) that were not related to the application site of FBT were somnolence (N = 9 [64.3%]), decreased oxygen saturation (N = 4 [28.6%]), headache (N = 3 [21.4%]), fatigue (N = 2 [14.3%]), nausea (N = 2 [14.3%]), and venipuncture-site pain (N = 2 [14.3%]). The 4 instances of decreased oxygen saturation, which were based on the clinical judgment of the investigator, were reported as mild and related to study drug, and all resolved with no action taken. The most frequently reported application-site AEs (incidence ≥10% of volunteers) were pain (N = 8 [57.1%]), erythema (N = 6 [42.9%]), and unspecified site reactions (N = 5 [35.7%]). In addition, there was 1 occurrence of each of the following application-site AEs: bleeding, irritation, ulcer, and vesicles. The incidence of changes in the oral mucosa increased in proportion to the number of doses administered. There were no clinically significant changes in clinical laboratory test results, vital sign values, ECG parameters, or physical examination findings during this study.

## Discussion

The primary objectives of this study were to characterize the pharmacokinetics of fentanyl, including its accumulation, and the safety and tolerability following administration of FBT 400 µg every 6 hours for a total of 10 doses. Accumulation of fentanyl was noted from doses 1 to 10, with systemic exposure (AUC_0–6_) 55% higher and C_max_ 23% higher after the tenth dose compared with the first dose. Steady state was reached within 3 days of dosing at 6-hour intervals, i.e., prior to dose 10. Fentanyl was rapidly absorbed following buccal administration of FBT 400 µg, with a median t_max_ of approximately 50 minutes following a single dose and 30 minutes after multiple doses.

Dwell time of FBT in the buccal mucosa varied from 9 to 60 minutes; however, it has been shown that the pharmacokinetic profile of fentanyl is not affected by the dwell time [12].

Fourteen volunteers had AEs, all of which were mild or moderate, with no serious AEs. The most common AEs not related to the application site of FBT were generally consistent with those previously reported in healthy volunteers who received FBT and naltrexone [5,6,8,13]. The 4 instances of decreased oxygen saturation were reported as mild and related to study drug, and all resolved with no action taken. The frequency of changes in the oral mucosa increased as the number of administered doses increased; therefore, patients should alternate dosing between the two sides of the mouth if they have mucosal irritation.

Limitations of this study include the enrollment of healthy volunteers, who may not represent a patient population. In particular, safety and tolerability findings would not be expected to reflect those in patients because the healthy volunteers in this study were administered oral naltrexone to minimize the opioid effects of fentanyl.

In conclusion, in healthy Japanese volunteers who received FBT 400 µg every 6 hours for 10 doses, fentanyl steady state was achieved prior to dose 10 (i.e., approximately 3 days of dosing at 6-hour intervals), and systemic exposure was 55% (AUC_0–6_) and 23% (C_max_) higher at steady state than after a single dose.
